# Chromosome-Level Genome Assembly of the Butter Clam *Saxidomus purpuratus*

**DOI:** 10.1093/gbe/evac106

**Published:** 2022-07-26

**Authors:** Jungeun Kim, Hui-Su Kim, Jae-Pil Choi, Min Sun Kim, Seonock Woo, Yeonghye Kim, Yejin Jo, Seungshic Yum, Jong Bhak

**Affiliations:** Personal Genomics Institute (PGI), Genome Research Foundation (GRF), Cheongju 28190, Republic of Korea; Korean Genomics Center (KOGIC), Ulsan National Institute of Science and Technology (UNIST), Ulsan 44919, Republic of Korea; Personal Genomics Institute (PGI), Genome Research Foundation (GRF), Cheongju 28190, Republic of Korea; Personal Genomics Institute (PGI), Genome Research Foundation (GRF), Cheongju 28190, Republic of Korea; Marine Biotechnology Research Center, Korea Institute of Ocean Science and Technology, Busan 49 111, Republic of Korea; Fisheries Resources Management Division, National Institute of Fisheries Science, Busan 46083, Republic of Korea; Ecological Risk Research Division, Korea Institute of Ocean Science and Technology (KIOST), Geoje 53201, Republic of Korea; Ecological Risk Research Division, Korea Institute of Ocean Science and Technology (KIOST), Geoje 53201, Republic of Korea; KIOST School, University of Science and Technology, Geoje 53201, Republic of Korea; Personal Genomics Institute (PGI), Genome Research Foundation (GRF), Cheongju 28190, Republic of Korea; Korean Genomics Center (KOGIC), Ulsan National Institute of Science and Technology (UNIST), Ulsan 44919, Republic of Korea; Department of Biomedical Engineering, School of Life Sciences, UNIST, Ulsan 44919, Republic of Korea; Clinomics, Inc., Ulsan 44919, Republic of Korea

**Keywords:** butter clam, *Saxidomus purpuratus*, genome, chromosome level

## Abstract

Herein, we provide the first whole-genome sequence of the purple butter clam (*Saxidomus purpuratus*), an economically important bivalve shellfish. Specifically, we sequenced and de novo assembled the genome of *Sa. purpuratus* based on PromethION long reads and Hi-C data. The 978-Mb genome of *Sa. purpuratus* comprises 19 chromosomes with 36,591 predicted protein-coding genes. The N50 length of *Sa. purpuratus* genome is 52 Mb, showing the highest continuous assembly among bivalve genomes. The Benchmarking by Universal Single-Copy Orthologs assessment indicated that 95.07% of complete metazoan universal single-copy orthologs (*n* = 954) were present in the assembly. Approximately 51% of *Sa. purpuratus* genome comprises repetitive sequences. Based on the high-quality *Sa. purpuratus* genome, we resolved half of the immune-associated genes, namely, scavenger receptor (SR) proteins, which are collinear to those in the closely related *Cyclina sinensis* genome. This finding suggested a high degree of conservation among immune-associated genes. Twenty-two (19%) SR proteins are tandemly duplicated in *Sa. purpuratus* genome, suggesting putative convergence evolution. Overall, *Sa. purpuratus* genome provides a new resource for the discovery of economically important traits and immune-response genes.

**Significance**

We performed chromosome-level genome assembly of *Saxidomus purpuratus*, the purple butter clam, and predicted approximately 36,591 protein-coding genes. We identified conserved and varied immune-response genes by comparing two high-quality clam genomes, *Sa. purpuratus* and *Cyclina sinensis*. This genome will facilitate further understanding of the genetic diversity and evolution of bivalves.

## Introduction

**Fig. 1.**
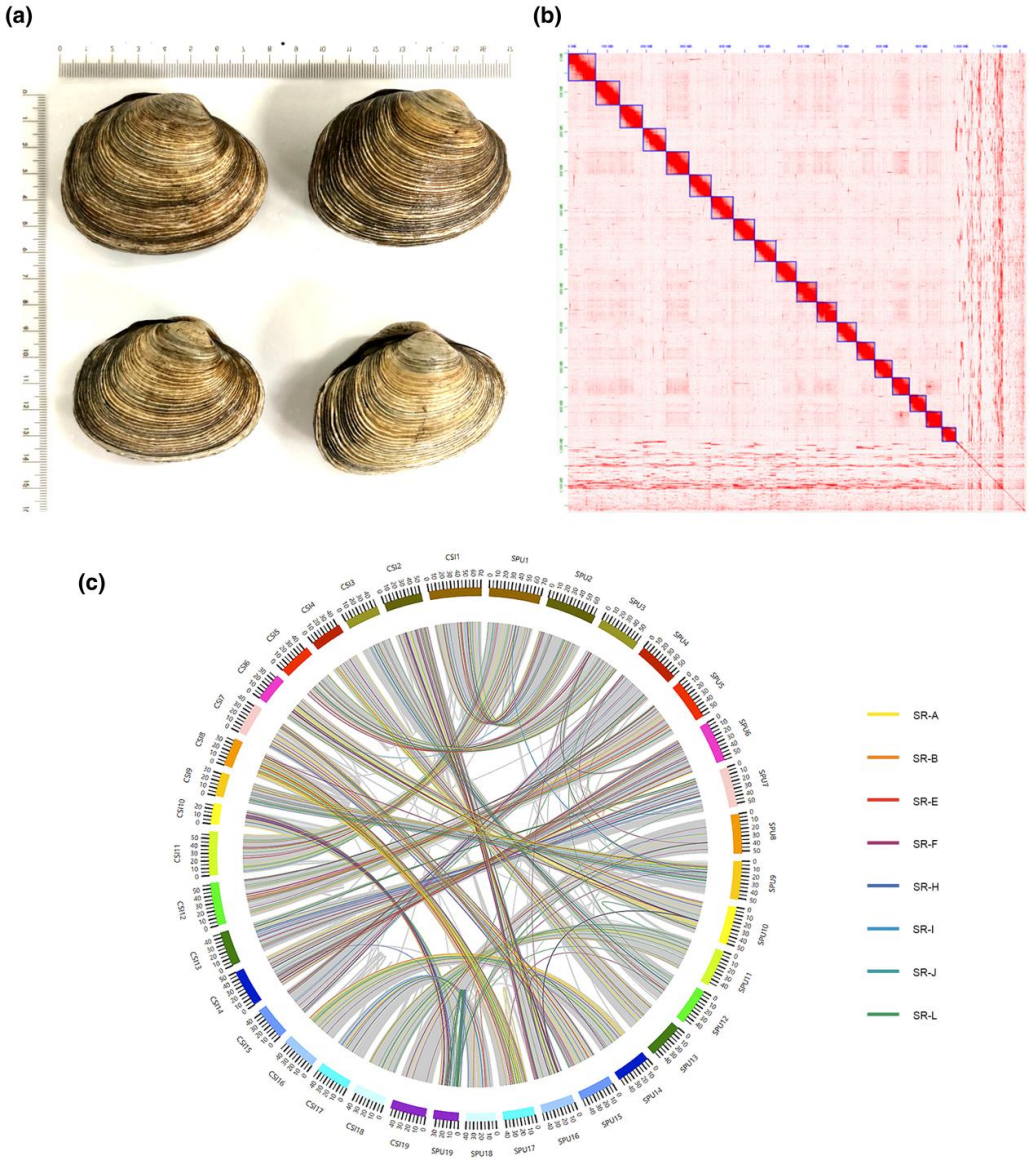
*Saxidomus purpuratus* and its genomic landscape. (*a*) *Saxidomus purpuratus* shells. (*b*). Hi-C contact map shows 19 pseudo-chromosomes of *Sa. purpuratus* genome. (*c*) Circus diagram represents collinear gene pairs (gray lines) between *Sa. purpuratus* (SPU) and *Cyclina sinensis* (CSI). Colored lines represent scavenger receptor (SR) proteins with evolutionary relationships predicted using MCScanX (Wang et al. 2012).

The purple butter clam (fig. 1*a*), *Saxidomus purpuratus* (NCBI: txid311201), is an economically important marine clam belonging to the family Veneridae, subclass Heterodonta, and class Bivalvia. Its habitat is mud up to 30-m deep in the intertidal zone of southwestern Korea (water temperature 3–26 °C, salinity: 30–33%). The shell of the purple butter clam is the heaviest and hardest compared with that of other Korean shellfish and is composed of outer calcite crystals and inner aragonite layers (Jiao et al. 2015). Previous omic studies have revealed the mitochondrial genome of *Sa. purpuratus* (Bao et al. 2016), as well as the transcriptome sequence for primary gene annotation and marker development (Li et al. 2017). In the current study, we generated the first whole-genome assembly of *Sa. purpuratus* and performed comparative genomic analysis, revealing that gene expansion is associated with adaptation to past marine chemical changes.

## Results and Discussion

### Genome Assembly of *Sa. purpuratus*

We sequenced 69.5 Gb of Illumina short reads and 250.3 Gb of PromethION long reads (supplementary table S1, Supplementary Material online) to construct a high-quality *Sa. purpuratus* reference genome (fig. 1*b*). Using 61 Gb of the cleaned short reads, GenomeScope estimated the genome size of *Sa. purpuratus* as 836 Mb with a high heterozygosity of 2.61% (supplementary fig. S1, Supplementary Material online). This estimate is within the 843 Mb (*Scapharca broughtonii*) to 1,071 Mb (*Ruditapes philippinarum*) range and is similar to previously assembled clam genomes (Bai et al. 2019; Yan et al. 2019; Wei et al. 2020). Based on the estimated genome size, our long and short reads covered 229- and 83-folds of *Sa. purpuratus* genome, respectively. To reduce the high heterozygosity, we assembled phased long reads and obtained a 1.06-Gb *Sa. purpuratus* assembly (table 1). For scaffolding, we sequenced 129.8 Gb of Hi-C reads and constructed 2,175 scaffolds (table 1). Finally, 19 pseudo-chromosomes were constructed with a 978-Mb genome, and the N50 length was 52 Mb (table 1, fig. 1*b*). The repeat contents accounted for 50.81% (497 Mb) of the assembly, of which 50.15% (490 Mb) are interspersed repeats and 43.00% are novel repeats (de novo identified repeats; supplementary table S2, Supplementary Material online). We predicted 37,690 protein-coding genes, 97.11% (36,591) of which were anchored to the 19 pseudo-chromosomes (table 1). We predicted the highest number of protein-coding genes relative to those of ten other bivalve genomes, except for the low-quality *Limnoperna fortunei* genome (Uliano-Silva et al. 2018; supplementary table S3, Supplementary Material online). In the Benchmarking by Universal Single-Copy Orthologs (BUSCO) assessment, we observed 96.6% completeness of the conserved core metazoan genes, including 95.1% of single-copy orthologs and 1.5% of duplicates (table 1). We observed the highest number of complete metazoan single-copy orthologs relative to ten other bivalve genomes and a relatively low number of complete duplicates (supplementary table S3, Supplementary Material online). This suggests a high-quality chromosome-level assembly of *Sa. purpuratus* genome.

**Table 1:** Statistics for *Saxidomus purpuratus* Genome Assembly

	Contigs	Scaffolds	Pseudo-chromosomes
No. of assembly	2,755	2,175	19
Total length	1,060,876,372	1,005,611,374	978,691,593
N50	1,696,621	52,399,280	52,399,280
Gap	0.000%	0.088%	0.090%
Max contig length	18,379,398	70,211,395	70,211,395
GC content	34.83%	34.83%	34.79%
No. of protein-coding genes		37,690	36,591
BUSCO (protein-coding genes)	C: 96.6% (S: 95.1%, D: 1.5%), F: 1.3%, M: 2.1%		
BUSCO (genome)	C: 94.2% [S: 93.5%, D: 0.7%], F: 3.6%, M: 2.2%		

### Phylogenomics and Gene Family Evolution

We analyzed genome conservation in Veneridae clams by comparing the high-quality genomes of *Sa. purpuratus* and *Cyclina sinensis* (Wei et al. 2020). We identified 14,771 collinear gene pairs in 4,019 syntenic blocks (fig. 1*c*), representing 12,824 (30.42%) *Sa. purpuratus* genes and 13,518 (49.04%) *C. sinensis* genes. We also analyzed the genome-wide distribution of scavenger receptor (SR) proteins (supplementary table S4, Supplementary Material online), which are known to be involved in the immune response of clams (Yan et al. 2019). We identified nine genes encoding SR family members from 38 orthologous groups (OGs), namely, *SR-A4*, *SR-A6*, *SR-B1*, *SR-E3*, *SR-F2*, *SR-H2*, *SR-I1*, *SR-L1*, and *SR-L2*, from an in-depth analysis. The SR proteins are distributed throughout bivalve genomes compared with those in gastropod (e.g., *Haliotis discus*) genomes (Nam et al. 2017). *SR-F2* is the most abundant SR family gene in bivalve genomes (supplementary table S4, Supplementary Material online). We examined *Sa. purpuratus* SR-protein-coding genes, which are collinear to those in *C. sinensis*. A total of 62 (53.45%) *Sa. purpuratus* SR proteins retained collinearity with closely related *C. sinensis* (fig. 1*c*). Genes encoding 22 (18.97%) *Sa. purpuratus* SR proteins and 19 (22.89%) *C. sinensis* SR proteins were tandemly duplicated in their genomes. In particular, three SR family genes, namely, *SR-A4*, *SR-L1*, and *SR-*L2, were observed to be expanded in the *Sa. purpuratus* genome. A previous study reported that SR-A4 induces an immune response by recognizing lipoproteins and oxidatively modifying low-density lipoproteins (Selman et al. 2008). Meanwhile, SR-L1 recognizes a myriad of cargo ligands or bioactive molecules (Herz and Strickland 2001), and SR-L2 binds to various internal ligands, including leptin, insulin, and amyloid peptide (Bartolome et al. 2017). In fact, mice lacking SR-L2 in brine endothelial cells exhibit neuroinflammation (Bartolome et al. 2017). Moreover, a previous functional study on SR proteins has revealed an association with the evolution of clam immunology, in particular, via recognition of a wide range of common ligands (Zani et al. 2015). Taken together, these results suggest that SR proteins have evolved independently in a specific lineage, which may explain both evolutionary consensus and divergence of SR proteins.

## Conclusion

The genome of *Sa. purpuratus*, the purple butter clam, comprises 19 pseudo-chromosomes with 36,591 protein-coding genes. Evolutionary comparison of the SR-protein-coding genes revealed the expansion of *SR-A4*, *SR-L1*, and *SR-L2* in *Sa. purpuratus* compared with those in other clam genomes. Half of the SR-protein-coding genes were collinear to *C. sinensis* genome, whereas 20% of them were randomly duplicated. Provision of this reference genome of an economically important bivalve shellfish could be a useful scientific resource for the genetic studies such as ecology and environmental adaptation.

## Materials and Methods

### Sample Collection and Genomic DNA

*Saxidomus purpuratus* samples were obtained from Eunpa Fisheries Company (Sadeung, Republic of Korea; juveniles, shell width of approximately 10 mm) and Jangmok Bay (Geoje, Gyeongnam, Republic of Korea; 34°59′21.2″N 128°40′52.4″E; adults, shell width of approximately 70 mm). The total DNA of *Sa. purpuratus* muscle tissue was extracted and processed as previously described (Kim et al. 2019).

RNA was extracted using 700 µl of water-saturated phenol. A 1/3 volume of 8 M LiCl was added to the retained aqueous phase, which was maintained at 4 °C for 2 h. RNA was precipitated after centrifugation at 16,000 × g for 30 min, followed by resuspension in 300 µl of diethylpyrocarbonate (DEPC)-treated water. Next, RNA was reprecipitated with 1/10 volume of 3 M sodium acetate (pH 5.2) and isopropanol. The precipitated RNA was rinsed with 70% ethanol (diluted in DEPC-treated water) and dissolved in an appropriate volume of DEPC-treated water (30–40 µl). The RNA library of *Sa. purpuratus* soft muscle was constructed using the Illumina TruSeq Stranded mRNA LT Sample Prep Kit (Illumina, Inc., San Diego, CA, USA) and sequenced on the NovaSeq 6000 platform (Macrogen, Inc., Seoul, Republic of Korea).

### Short-Read Sequencing and Genome Size Estimation

For short reads, DNA libraries were constructed using the TruSeq Nano HT Sample Preparation Kit (Illumina, Inc.), and paired-end reads were generated on the NovaSeq 6000 platform (Illumina, Inc.) according to the manufacturer’s instructions. For quality control of the short reads, we trimmed adapters and low-quality reads (*Q* < 20) using Trimmomatic (ver. 0.64; RRID: SCR_011848; Bolger et al. 2014). Prokaryotic contaminant reads were removed using the bbsplit.sh script in Bbtools (ver. 38.26; https://jgi.doe.gov/data-and-tools/bbtools; RRID: SCR_016965) and NCBI prokaryotic RefSeq genomic database.

We estimated *Sa. purpuratus* genome size using the trimmed short reads with Jellyfish (ver. 2.2.4; RRID: SCR_005491; Marcais and Kingsford 2011) and GenomeScope (ver. 2; RRID: SCR_017014; Ranallo-Benavidez et al. 2020). Jellyfish was used to calculate the *K*-mer frequency with 21-mer readouts, and GenomeScope was used to estimate *Sa. purpuratus* genome size (supplementary fig. S1, Supplementary Material online).

### Nanopore-based PromethION Long-Read Sequencing

Purified DNA was shredded into 40-kb fragments using a Covaris g-tube (Covaris, Inc., Woburn, MA, USA). Three genomic DNA libraries for *Sa. purpuratus* were constructed for PremethION sequencing using the ONT 1D ligation Sequencing Kit (SQK-LSK109; ONT, Oxford Nanopore Technologies, Oxford, UK), flow cell priming kit (EXP-FLP001.PR0.6), and PromethION R9.4.1 flow cell (FLO-PRO002) according to the manufacturer’s instructions. We generated FASTQ data using base calling with Guppy (ver. 5.0.11) and the CFR model (Wick et al. 2019), and used Porechop (ver. 0.2.4; https://github.com/rrwick/Porechop; RRID: SCR_016967) to remove adapter sequences and low-quality reads (quality score < 10) from the raw reads during the base-calling step.

### Hi-C Long-Range Mapping-based Data Generation and Sequencing

To construct an Hi-C library, we collected *Sa. purpuratus* muscle tissues from the same individuals used for long- and short-read sequencing. The Arima-Hi-C kit (Arima Genomics, Inc., San Diego, CA, USA) was used according to the manufacturer’s instructions. The Hi-C library was sequenced using the NovaSeq 6000 platform.

### Genome Assembly and Error Correction

To assemble *Sa. purpuratus* genome, we constructed a genome assembly pipeline with three steps of a draft assembly, read-based phasing, and a main assembly (supplementary fig. S2, Supplementary Material online). First, we assembled a draft assembly with PromethION long reads using Flye assembler (ver. 2.9; RRID: SCR_017016; Kolmogorov et al. 2019) and performed error correction using Medaka (ver. 0.8.1; https://github.com/nanoporetech/medaka) to infer base errors from contigs with the “CRF” model. To remove redundant contigs, we performed Purge Dups (ver. 1.2.5; https://github.com/dfguan/purge_dups; RRID: SCR_021173). Scaffolding contigs were initiated using Hi-C reads with Juicer (ver. 1.6.2; https://github.com/aidenlab/juicer; RRID: SCR_017226) and 3D-DNA pipeline (current release 180922; RRID: SCR_017227; Dudchenko et al. 2017).

As the second step, we performed variant calling against the draft assembly with long reads using the PEPPER-Margin-DeepVariant pipeline (ver. 0.6; Shafin et al. 2021) and read-based phasing using WhatsHap (ver. 1.1; Patterson et al. 2015). Reads with phased vcf were partitioned using WhatsHap with the command “whatshap split –discard-unknown-reads –pigz –output-h1 output.hap1 –output-h2 output.hap2 –output-untagged output.un –read-lengths-histogram output.hist phased.bam phased.tags.”

As the third step for main assembly, we assembled contigs with phased reads from hap1 using the Flye assembler. The error correction step was initiated in the same way as the draft assembly. To remove redundant contigs, HaploMerger2 (ver. 20180603; Huang et al. 2017) was used with the masked contig assembly. We constructed a repeat library using RepeatModeler (ver. 2.0; RRID: SCR_015027; Flynn et al. 2020) and masked repetitive sequences using RepeatMasker (ver. 4.1.2-p1; http://www.repeatmasker.org/RepeatMasker; RRID: SCR_012954; supplementary table S2, Supplementary Material online). Scaffolding contigs were initiated with Hi-C reads using Juicer and 3D-DNA pipeline. We finally constructed 19 pseudo-chromosomes by manual curation of misassemblies and redundant contigs using Juicebox Assembly Tools (ver. 1.13.01; RRID: SCR_021172; https://github.com/aidenlab/Juicebox). Single-nucleotide polymorphisms and indel errors on contigs were corrected by two rounds of polishing using Pilon (ver. 1.23; RRID: SCR_014731; Walker et al. 2014).

Finally, we constructed a 1.05-Gb assembly of 2,175 scaffolds with an N50 of 52.4 bp. Nineteen super-scaffolds (pseudo-chromosomes) represented the near chromosome-level assembly of *Sa. purpuratus* genome (table 1, fig. 1*b*).

### De novo Assembly of RNA-sequencing Data

Quality control of the RNA-sequencing reads was achieved by trimming adapter sequences and low-quality reads below a Phred-score of 20. Contaminated reads were removed as described for the genomic short reads. De novo assembly of the transcriptome was performed using Trinity assembler (ver. 2.11.0; RRID: SCR_013048; Grabherr et al. 2011). Finally, we extracted coding regions within the assembled transcripts using TransDecoder (ver. 5.3.0; RRID: SCR_017647; https://github.com/TransDecoder/TransDecoder/).

### Gene Prediction

To predict protein-coding genes, we prepared a two-pass pipeline using BRAKER2 (ver. 2.1.5; RRID: SCR_018964; Bruna et al. 2020) and TSEBRA (ver. 1.0.3; Gabriel et al. 2021). For accurate gene prediction, we aligned the RNA-sequencing reads using STAR aligner (ver. 2.7.8a; RRID: SCR_015899; Dobin et al. 2013) and protein sequences of molluscan OrthoDB (ver. 10.1; RRID: SCR_011980; Simao et al. 2015) with ProHint (ver. 2.6.0; https://github.com/gatech-genemark/ProtHint). First, two iterations of the BRAKER2 pipeline with mapped RNA-sequencing data and the molluscan OrthoDB sequences were performed sequentially. The best gene models were selected from the predicted gene models using the TSEBRA pipeline with default parameters.

We assessed *Sa. purpuratus* genome using the BUSCO analysis with molluscan OrthoDB (ver. 5.2.1) and compared the BUSCO values with those of ten bivalve genomes, including two scallops (Atlantic bay scallop [*Argopecten irradiansr*; Liu et al. 2020]; and bay scallop [*A. purpuratus*; Li et al. 2018]), two mussels (golden mussel [*L. fortunei*; Uliano-Silva et al. 2018] and deep-sea mussel [*Bathymodiolus platifrons*; Wong et al. 2015]), two oysters (pearl oyster [*Pinctada fucata*; Takeuchi et al. 2016] and Black-shelled Pacific Oyster [*Crassostrea gigas*; Wang et al. 2019]), and four clams (snout otter clam [*Lutraria rhynchaena*; Thai et al. 2019]; blood clam [*Sc. broughtonii*; Bai et al. 2019]; Manila clam [*R. philippinarum*; Yan et al. 2019], and black clam [*C. sinensis*; Wei et al. 2020]) genomes (supplementary table S3, Supplementary Material online). One gastropod (*H. discus*) genome was used as the outgroup (Nam et al. 2017).

### Gene Annotation

Gene annotation was performed using BLASTp (RRID: SCR_004870; Altschul et al. 1990) against the SWISS-PROT database (ver. 2020_06; RRID: SCR_004426; Bairoch and Apweiler 2000), and domains were identified using InterProScan (ver. 5.36-75; RRID: SCR_005829; supplementary table S3, Supplementary Material online; Jones et al. 2014). The domains of SR proteins were identified based on Pfam (ver. 32; RRID: SCR_004726) domains from the InterProScan results.

### Orthologous Gene Family and Synteny Analysis

For effective comparative analysis, representative bivalve genomes with a high N50 were selected and analyzed using long-read-based assembly (supplementary table S3, Supplementary Material online). We collected ten bivalve genomes including those of two scallops, two oysters, and four clams.

We defined orthologous relationships using OrthoMCL (ver. 2.0.9; RRID: SCR_007839; Li et al. 2003). To identify the synteny blocks, we downloaded the gff file of *C. sinensis* from Dryad Data web (https://datadryad.org/stash/dataset/doi:10.5061/dryad.44j0zpcb5). Collinear gene pairs between *Sa. purpuratus* and *C. sinensis* were analyzed using the MCScanX toolkit (Wang et al. 2012).

### Classification of Scavenger Receptors

We collected 66 previously classified SR proteins in humans and mice (supplementary table S5, Supplementary Material online; Zani et al. 2015) and identified their domains using Pfam (supplementary table S5, Supplementary Material online). These data were used to classify the SR proteins in our samples. The protein sequences were subjected to homology searches against human and mouse SR proteins (*e*-value < 1*e*^-10^), and SR-coding domains were identified. We mapped putative SR proteins in the OG (supplementary table S4, Supplementary Material online). Considering that several orthologous genes classified by the OrthoMCL algorithm were found to lack the SR-coding domain, we defined an SR-protein OG when >50% of the protein members conserved the SR-coding domain and more than ten OG members were included. Based on these criteria, we identified 38 OGs for nine SR-protein families and manually identified species-specific expansion of the SR proteins in each OG.

## Supplementary Material

evac106_Supplementary_Data

## Data Availability

All sequences generated in this study, including PromethION long reads and Illumina short reads, have been deposited in the NCBI SRA under BioProject PRJNA706842. The genome assembly and annotation files are available under acc. GCA_022818135.1.
