# Supplementary material for: Chromosome-Level Genome Assembly of the Butter Clam Saxidomus purpuratus
Source: Genome Biol Evol. 2022 Jul 26;14(7):evac106. doi: 10.1093/gbe/evac106 (PMC9337622; doi:10.1093/gbe/evac106)
Supplement: evac106_Supplementary_Data [file evac106_supplementary_data.zip › Supplementary_figures_20220620.docx]

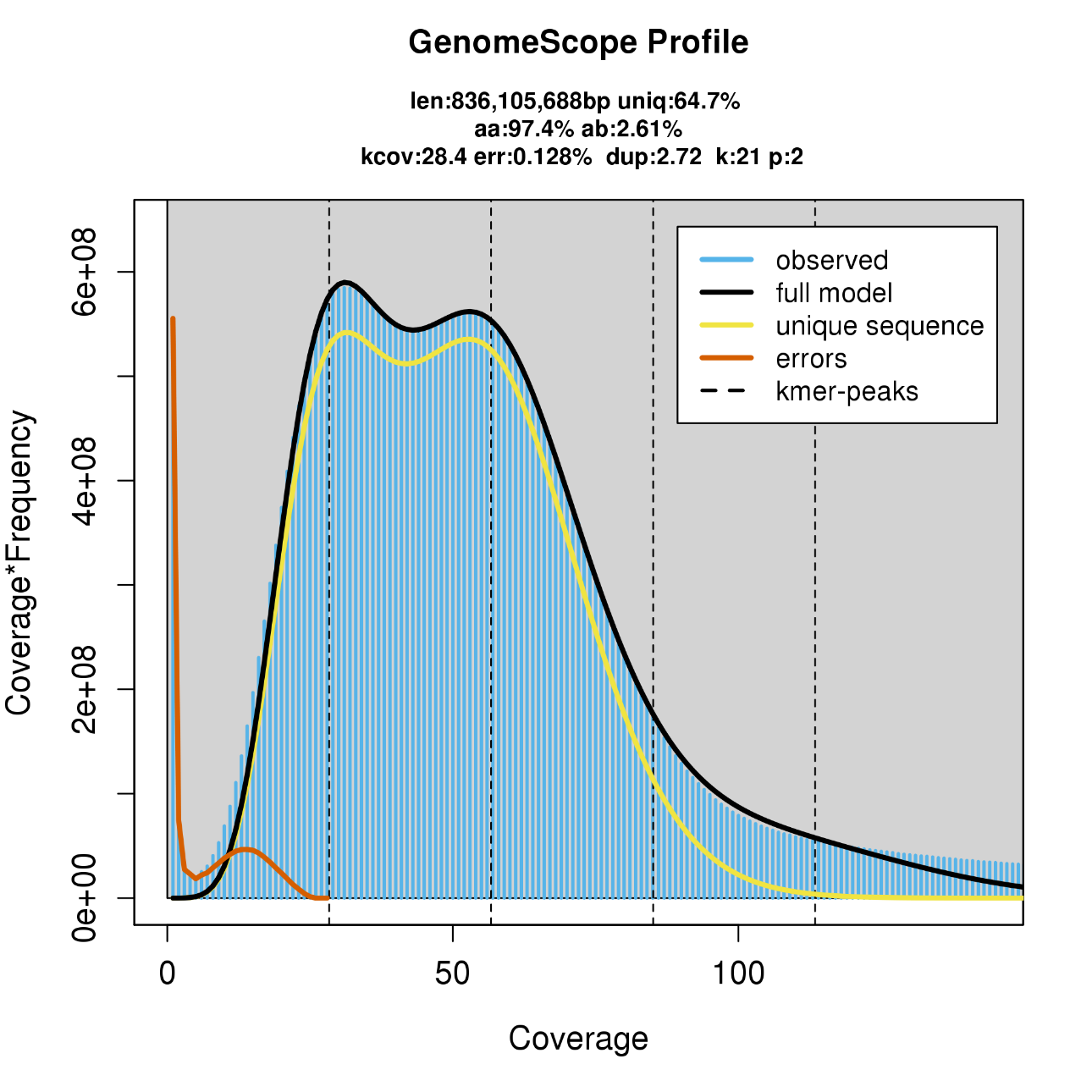


Supplementary Figure S1. Estimation of genome size for *Saxidomus purpuratus*

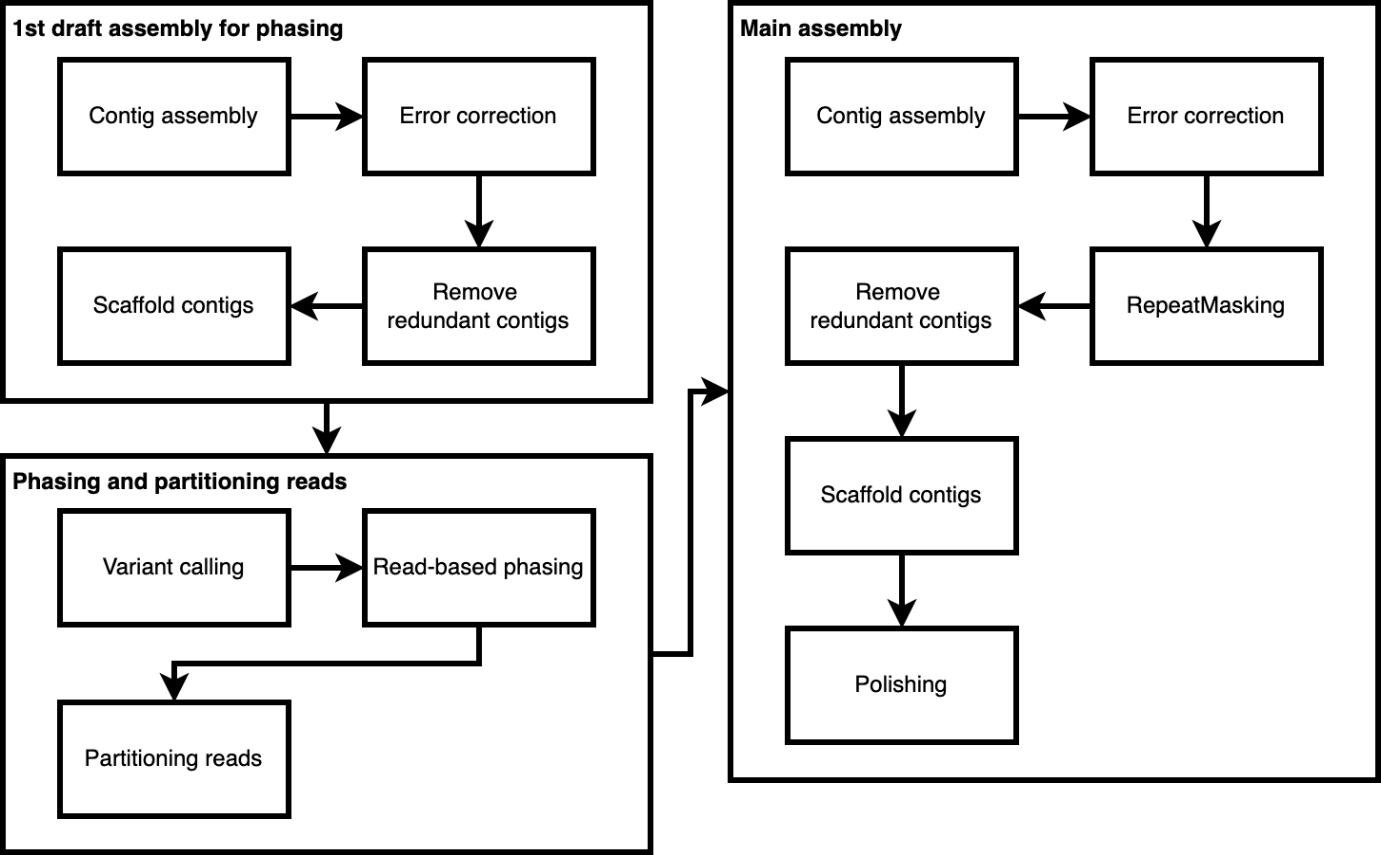


Supplementary Figure S2. *de novo* assembly pipeline for *S. purpuratus* genome
